# Curcumol Induces G1 Phase Arrest in SK-Hep-1 Cells by Targeting SKP2-Mediated p27 Degradation

**DOI:** 10.3390/molecules31060997

**Published:** 2026-03-16

**Authors:** Yizhuang Yang, Riqiu Zhang, Tong Dou, Zhangchi Liu, Rui Ai, Yue Zhao, Zhi Cui, Xu Chen, Juan Wang

**Affiliations:** 1 Department of Pharmacy, Guilin Medical University, Zhiyuan Road, Lingui, Guilin 541199, China; yyz8018@163.com (Y.Y.);; 2 Faculty of Basic Medicine, Guilin Medical University, Zhiyuan Road, Lingui, Guilin 541199, China; 3 School of Pharmacy, Macau University of Science and Technology, Avenida Wai Long, Macau 999078, China

**Keywords:** curcumol, hepatocellular carcinoma, cell cycle, SKP2, p27

## Abstract

Context: S-phase kinase-associated protein 2 (SKP2) is an oncogene and cell cycle regulator that mediates the ubiquitination of cell cycle regulators. Curcumol, a sesquiterpene natural product, has been reported to regulate SKP2-mediated ubiquitination degradation to overcome drug resistance in cancer cells. However, whether the cell cycle arrest effect of curcumol is related to SKP2’s function in cancer cells and its mechanisms are still unclear. Objective: To investigate the role of SKP2 in curcumol-induced cell cycle arrest and its underlying mechanisms. Materials and Methods: Transcriptomic and proteomic analyses were used to screen the ubiquitination-related factors in curcumol treated hepatocellular carcinoma cells. Lentiviral overexpression, co-immunoprecipitation assays, ubiquitination analysis, and cell-line-derived xenograft (CDX) models were used to dissect the role and mechanisms of the identified ubiquitination-related factor in the cell cycle arrest effect of curcucmol. Results: Curcumol modulated the expression of CDK4, CDK6, Cyclin D1, p27 and SKP2. SKP2 was one candidate target of curcumol selected by multi-omics. Overexpressed SKP2 partially reversed curcumol-induced growth inhibition and G1-phase arrest. The increased expression of p27 induced by curcumol was attenuated by overexpressed SKP2. Curcumol impaired the interaction between SKP2 and p27, and led to the ubiquitination and degradation of p27. In vivo, curcumol effectively reduced tumor growth, and its antitumor effect was significantly mitigated by SKP2 overexpression. Discussion and Conclusions: Curcumol reduced SKP2 expression, weakened the interaction between SKP2 and p27, inhibited degradation of p27, and then induced G1 phase cell-cycle arrest in SK-Hep-1 cells.

## 1. Introduction

Liver cancer is the third leading cause of cancer-related deaths worldwide [1]. Hepatocellular carcinoma (HCC) accounts for about 90% of liver cancer cases [2]. Despite advances in innovative therapies, including cellular immunotherapy, tumor ablation, and nanoparticle-based drug delivery, these treatments still face significant challenges such as drug resistance, limited efficacy, and adverse side effects [3,4,5]. Therefore, a better understanding of the mechanisms of HCC, or finding novel drugs with lower toxicity will have a high clinical impact [6].

*Curcuma zedoaria* (Christm.) Rosc. is a traditional Chinese medicinal herb [7]. It is derived from the dried rhizomes of *Curcuma phaeocaulis*, *Curcuma kwangsiensis* S. G. Lee & C. F. Liang, or *Curcuma wenyujin* Y. H. Chen & C. Ling, with *Curcuma kwangsiensis* S. G. Lee & C. F. Liang recognized as a locally sourced medicinal material in Guangxi. One of its major bioactive components, curcumol, is a sesquiterpene extracted from its volatile oil and has been reported to show obvious anti-tumor activity [8]. Our previous studies demonstrated that curcumol exhibited substantial proliferation inhibition on the HCC cells, and SK-Hep-1 cells showed the most sensitivity to its effect. Meanwhile, curcumol was found to be less toxic to normal hepatocytes [9]. Furthermore, we found that curcumol can induce G1 phase arrest in SK-Hep-1 cells and PLK1 may not be the direct target of curcumol-induced G1 phase arrest [9].

Regulation of the G1/S transition depends on the activation of cyclin-dependent kinases (CDKs), whose activities are regulated by the ubiquitin-mediated proteolysis of cyclins and CDK inhibitors (CKIs). SKP2 is a key F-box protein of the SCF complex that mediates the ubiquitination and degradation of multiple substrates [10]. One of its key targets is P27Kip1 (p27), a cyclin-dependent kinase (CDK) inhibitor encoded by CDKN1B, which suppresses CDK4/6 activity to regulate the G1/S transition [11,12]. In HCC, SKP2 is frequently overexpressed, leading to excessive ubiquitination and degradation of p27. This dysregulation compromises G1/S checkpoint control, promotes uncontrolled cell proliferation, and is associated with poor prognosis [13,14]. Our previous study found SKP2 and PLK1 to be candidate targets of curcumol in HCC [9], but PLK1 mainly regulates the G2/M transition [15], with only limited evidence linking it to p53-mediated G1 arrest [16]. We therefore considered curcumol might also induce G1-phase arrest through other pathways.

In this study, we aim to elucidate the role of SKP2 in curcumol-induced cell cycle arrest and its underlying mechanisms in vitro and in vivo, and to define the critical role of the SKP2–p27 axis in mediating its therapeutic effects.

## 2. Results

### 2.1. Curcumol-Induced G1 Phase Arrest in SK-Hep-1 Cells

**Figure 1:**
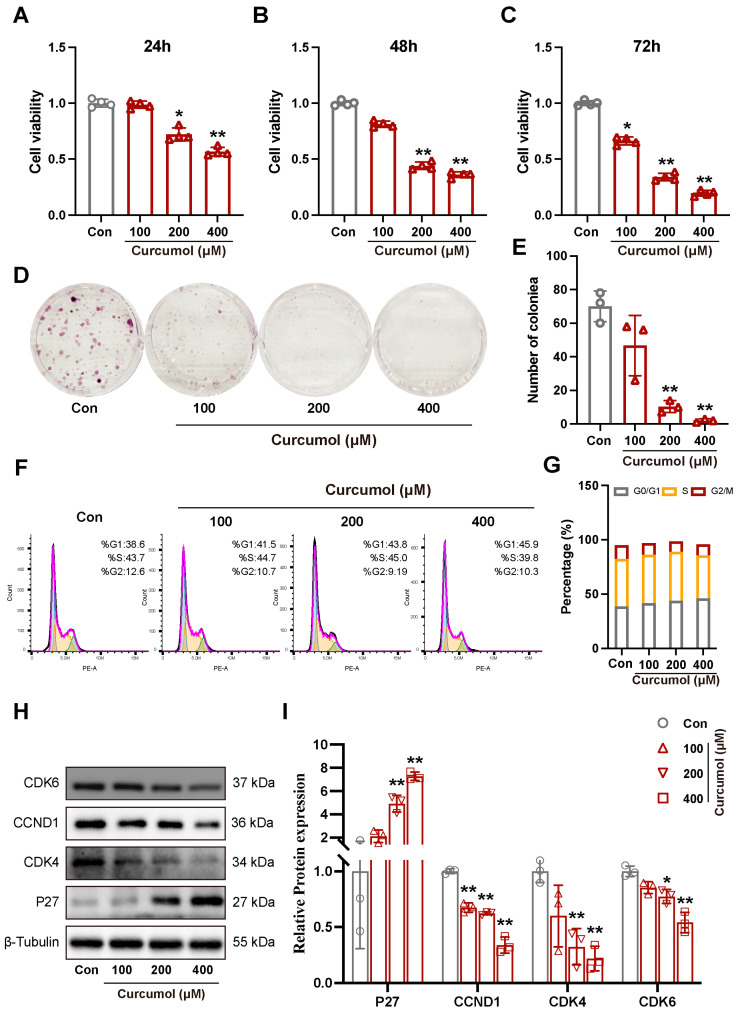
Curcumol induces G1 cell cycle arrest in SK-Hep-1 cells. (**A**–**C**) Curcumol inhibited the proliferation of SK-Hep-1 cells. (**D**,**E**) Colony formation assays in SK-Hep-1 cells with and without curcumol treatment. (**F**,**G**) The effect of curcumol treatment on the cell cycle distribution of SK-Hep-1 cells is analyzed. (**H**) Immunoblot analysis of p27, CCND1, CDK4, and CDK6 in SK-Hep-1 cells after treatment with curcumol. (**I**) Densitometric analysis of (**H**). All data are presented as means ± SD, *n* = 3. Compared with the Con group, * *p* < 0.05, ** *p* < 0.01.

The results of the MTT experiments showed that curcumol could decrease the cell viability of SK-Hep-1 cells (Figure 1A–C). In addition, treatment of SK-Hep-1 cells with curcumol inhibited colony formation (Figure 1D,E). Furthermore, curcumol significantly arrested SK-Hep-1 cells in the G1 phase (Figure 1F,G), which is consist with our previous report [9]. Western blot results showed that the expression of Cyclin D1, CDK4, and CDK6 were significantly downregulated, and the expression of p27 was significantly upregulated after curcumol treatment (Figure 1H,I).

### 2.2. Integrated Transcriptomic and Proteomic Analyses Identified SKP2 as a Potential Regulator of Curcumol-Induced G1 Arrest

**Figure 2:**
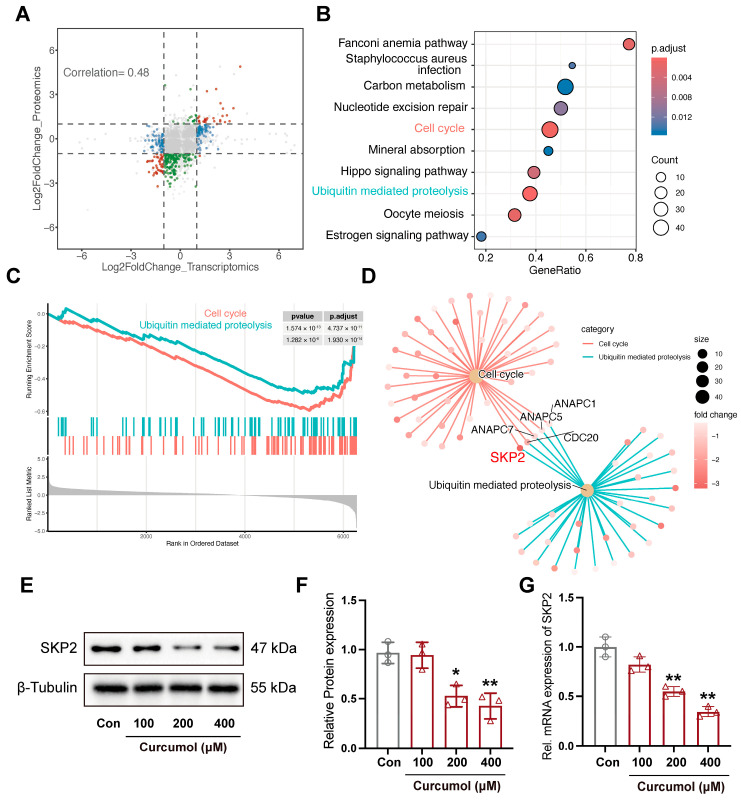
Identification of SKP2 as a potential regulator of curcumol-induced G1 arrest. (**A**) The scatter plot showed the correlation between the transcriptomic and proteomic data. (**B**) KEGG pathway enrichment analysis based on proteomic data. (**C**) GSEA of the significantly enriched KEGG pathways related to cell cycle and ubiquitin-mediated proteolysis, performed based on the proteomic dataset. (**D**) The network visualization showed the common proteins between the cell cycle and ubiquitin-mediated proteolysis pathways, derived from the proteomic-based GSEA results. (**E**) Immunoblot analysis of SKP2 in SK-Hep-1 cells after treatment with curcumol. (**F**) Densitometric analysis of (**E**). (**G**) mRNA expression levels of SKP2. All data are presented as means ± SD, *n* = 3. Compared with the Con group, * *p* < 0.05, ** *p* < 0.01.

The scatter plots show mRNA and protein level changes after curcumol treatment; the Pearson correlation coefficient was 0.48 (Figure 2A). As shown in Figure 2B, differentially expressed proteins were found to be enriched in cell cycle and ubiquitin-mediated proteolysis. GSEA revealed a reduction in cell cycle and ubiquitin-mediated proteolysis pathways in the curcumol-treated group (Figure 2C). SKP2, CDC20, ANAPC7, ANAPC5, and ANAPC1 are common genes involved in both the cell cycle and ubiquitin-mediated pathways. Our previous study using a different analytical approach also suggested SKP2 as a potential target of curcumol [9], and SKP2 exhibited the most significant reduction in protein expression levels following curcumol treatment in SK-Hep-1 cells (Figure 2D). Western blot analyses and qRT-PCR validated that curcumol treatment significantly reduced the mRNA and protein expression of SKP2 (Figure 2E,F).

### 2.3. Overexpression of SKP2 Rescues SK-Hep-1 Cells from Curcumol-Induced Proliferation Inhibition and G1 Arrest

**Figure 3:**
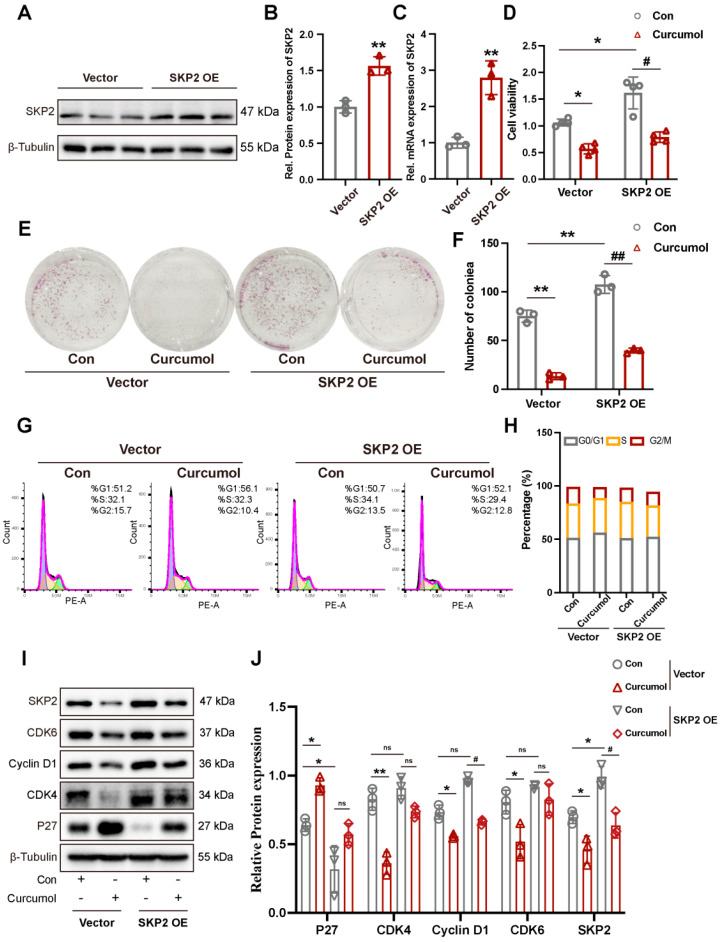
SKP2 overexpression reduces curcumol-induced G1 arrests. (**A**) Immunoblot analysis of SKP2. (**B**) Densitometric analysis of (**A**). (**C**) mRNA expression levels of SKP2. (**D**) Cell viability assessed by MTT assay. (**E**,**F**) Colony formation assays were performed on SK-Hep-1 cells stably expressing SKP2 with curcumol treatment. (**G**,**H**) The effect of curcumol treatment on the cell cycle distribution of SK-Hep-1 cells stably expressing SKP2 was analyzed. (**I**) Immunoblot analysis of p27, CCND1, CDK4, CDK6, and SKP2. (**J**) Densitometric analysis of (**I**). All data are presented as means ± SD, *n* = 3. Compared with the Vector-Con group, * *p* < 0.05, ** *p* < 0.01; compared with the SKP2 OE-Con group, ^#^ *p* < 0.05, ^##^ *p* < 0.01.

To investigate the role of SKP2 in curcumol-induced G1 block in SK-Hep-1 cells, we used lentiviral transfection to construct SK-Hep-1 cell lines stably overexpressing SKP2. Western blot and qRT-PCR confirmed significant upregulation of SKP2 at both the mRNA and protein levels (Figure 3A–C). MTT and colony formation assays showed that SKP2 overexpression significantly enhanced SK-Hep-1 cell viability and colony formation (Figure 3D–F). SKP2 overexpression also attenuated curcumol’s inhibitory effects on SK-Hep-1 cells’ proliferation and colony formation (Figure 3D–F). Flow cytometry analysis revealed that SKP2 overexpression significantly attenuated curcumol-induced G1 phase arrest in SK-Hep-1 cells (Figure 3G,H). Western blot analysis further demonstrated that SKP2 overexpression reversed curcumol’s regulated effects on CDK4, CDK6, and Cyclin D1 and p27 (Figure 3I,J).

### 2.4. Curcumol-Induced Reduction of p27 Ubiquitination Is Dependent on SKP2

**Figure 4:**
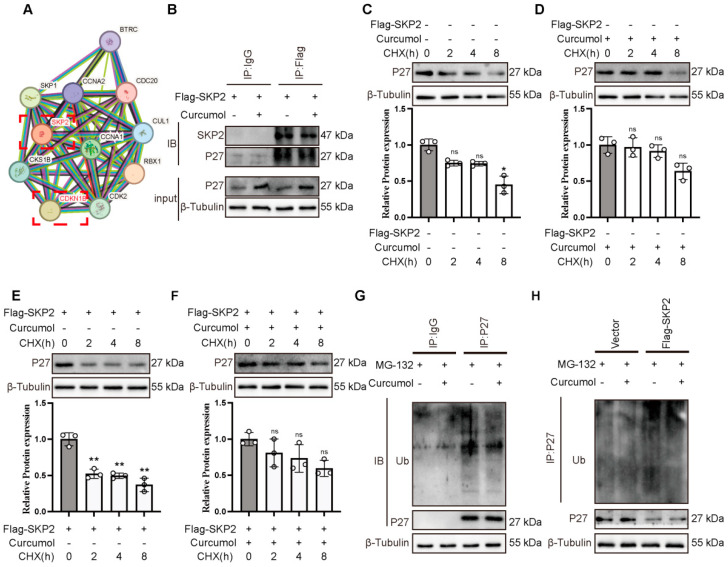
Curcumol inhibits p27 ubiquitination and degradation in SK-Hep-1 cells. (**A**) The STRING protein–protein interaction network of SKP2. (**B**) Co-IP analysis of SK-Hep-1 cells with Curcumol treatment for 48 h. (**C**–**F**) Immunoblot analysis of SK-Hep-1 cells with CHX treatment for indicated times following treatment with curcumol. (**G**) SK-Hep-1 cells were treated with Curcumol for 48 h, then with MG132, and finally subjected to ubiquitination analysis. (**H**) SK-Hep-1 cells were transfected with control or SKP2 overexpression vectors treated with curcumol for 48 h then with MG132 and finally subjected to ubiquitination analysis. All data are presented as means ± SD, *n* = 3. Compared with the corresponding 0 h group within the same experimental condition: * *p* < 0.05, ** *p* < 0.01.

SKP2-mediated ubiquitylation and degradation of p27 are important steps for the G1/S phase transition [10,17]. We used interaction databases (https://string-db.org/) and identified CDKN1B (p27) as a potential interacting partner of SKP2 (Figure 4A). Co-IP analysis confirmed that p27 interacted with SKP2 in SK-Hep-1 cells, and this interaction was reduced upon curcumol’s treatment (Figure 4B). Moreover, curcumol prolonged the half-life of endogenous p27 in the presence of CHX, whereas this effect was attenuated by the overexpression of SKP2 in SK-Hep-1 cells (Figure 4C–F). Consistently, ubiquitination analysis showed that curcumol decreased the endogenous polyubiquitination of p27 (Figure 4G). Overexpression of SKP2 restored the p27 ubiquitination that was reduced by curcumol (Figure 4H).

### 2.5. Curcumol Inhibited Tumor Growth in Mice by Inhibiting SKP2 Expression

**Figure 5:**
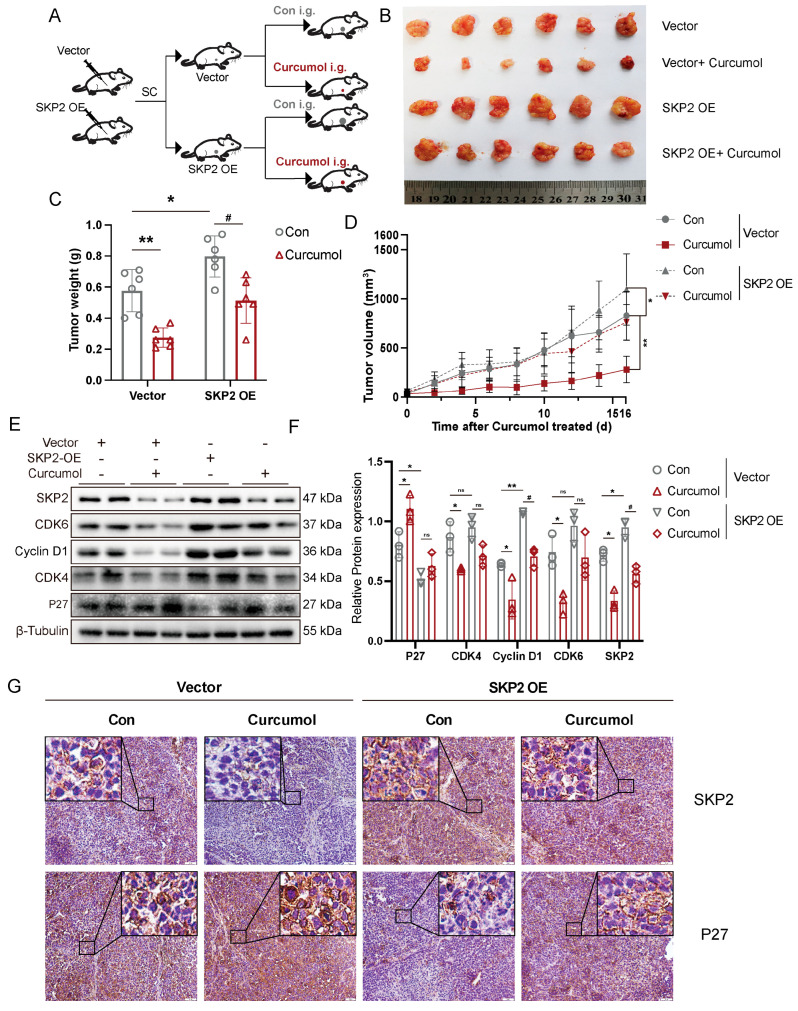
SKP2 overexpression attenuates the inhibitory effect of curcumol on tumor growth in mice. (**A**) A subcutaneous xenograft model was established by subcutaneously injecting cells overexpressing SKP2 or transfected with a vector control. Subsequently, the experimental animals were treated with curcumol via intragastric administration. (**B**–**D**) The mass (**B**), weight (**C**) and volume (**D**) of SK-Hep-1 xenografts generated from control or SKP2-overexpressing cells following treatment with vehicle control or curcumol. (**E**) Immunoblot analysis of p27, CCND1, CDK4, CDK6, and SKP2. (**F**) Densitometric analysis of (**E**). (**G**) The expression of SKP2 and p27 in the tissues was detected by Immunohistochemical staining, Bar: 50 μm. All data are presented as mean ± standard deviation (SD), *n* = 6. Compared with the Vector-Con group, * *p* < 0.05, ** *p* < 0.01; compared with the SKP2 OE-Con group, ^#^ *p* < 0.05.

We next investigated the role of SKP2 in the cell cycle progression in vivo using a curcumol-treated xenograft model (Figure 5A). We found that curcumol exhibited a significant inhibitory effect on SK-Hep-1 xenograft tumors. Moreover, the average tumor weight of xenograft tumors treated with curcumol was significantly reduced compared to that of the vehicle-treated groups. Overexpression of SKP2 significantly increased tumor volumes and weights compared to the Vector group, the inhibitory effect of curcumol on tumor volumes and weights was attenuated by SKP2 overexpression (Figure 5B–D). Western blot analysis showed that SKP2 overexpression effectively reversed the changes in G1 regulatory protein expression caused by curcumol (Figure 5E,F). Immunohistochemistry staining showed that curcumol treatment downregulated the expression of SKP2 and upregulated the expression of p27. However, this effect of curcumol was attenuated by SKP2 overexpression (Figure 5G).

## 3. Discussion

HCC is one of the most common malignant tumors globally, with its high incidence and mortality rates highlighting its significance as a major public health concern. Standard treatments, including surgical resection, chemotherapy and targeted therapy, can partially control disease progression, yet their effectiveness is constrained by challenges such as frequent recurrence and drug resistance. As a result, identifying novel therapeutic strategies has become a pressing research priority. The development and progression of HCC is characterized by a complex, multifactorial process marked by the dysregulation of multiple molecular pathways. Notably, aberrant cell cycle regulation is a key pathological feature of HCC. The G1/S transition, a critical checkpoint regulating the shift from G1 to S phase, plays an essential role in cell cycle control. In HCC, disruption of this transition promotes excessive tumor cell proliferation, exacerbating disease progression. Thus, targeting the regulation of the G1/S transition represents a promising approach for advancing HCC therapeutic research [18,19]. SKP2 is an extensively investigated E3 ubiquitin ligase that regulates the cell cycle. It facilitates cell cycle progression by targeting the degradation of cell cycle-dependent kinase inhibitors, such as p27 [20], thereby alleviating the constraints on the cell cycle and promoting the transition from the G1 phase to the S phase. Numerous studies have demonstrated that SKP2 is markedly upregulated in multiple tumors as an oncogene and plays a pivotal role in tumor initiation and progression [21,22]. Consequently, the identification and optimization of drugs targeting SKP2 are regarded as promising directions for tumor therapy.

**Figure 6:**
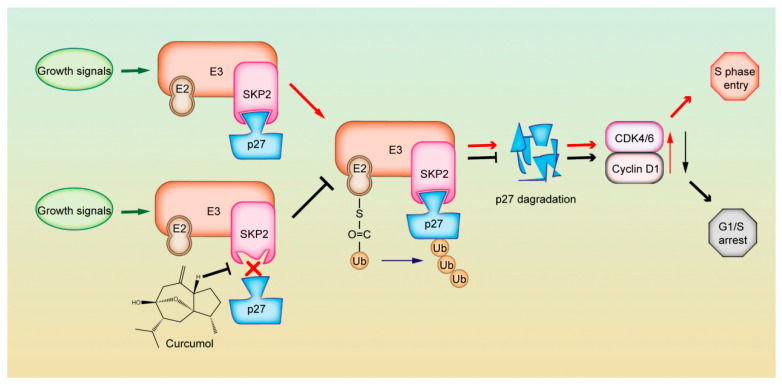
Curcumol induces G1 phase arrest in SK-Hep-1 cells via modulating SKP2-mediated p27 degradation.

In this study, we found that curcumol significantly inhibited SK-Hep-1 cell proliferation and induced G1 phase arrest, blocking the cancer cells’ entry into the S phase. Multi-omics and functional analyses revealed cell cycle and ubiquitin-mediated proteolysis pathways may be the candidate pathways influenced by curcumol, and SKP2 was chosen as the key factor regulating the two pathways via transcriptomic and proteomic integrated analyses. Overexpression of SKP2 is frequently observed in human malignancies. It regulates phosphorylated cell cycle regulator proteins and their ubiquitination as a cell cycle regulator and oncogene [23,24]. It has been reported that SKP2 is often overexpressed in HCC and is associated with low p27 levels, dysregulated G1/S transition and poor prognosis [18]. In this study, we found that curcumol inhibited the expression of SKP2 in HCC. Moreover, we found that SKP2 overexpression reduced curcumol-mediated G1 phase arrest in HCC and decreased the upregulated effect of curcumol on p27. p27 is a critical substrate of SKP2. Loss of SKP2 resulted in stabilization of p27, its main substrate for ubiquitination and subsequent proteasomal degradation, proteasomal degradation of p27 protein is an important method of p27 regulation [25]. Wang et al. found simvastatin inhibits STAT3 signaling to reduce SKP2 transcription, stabilizing p27 and inducing G0/G1 arrest [13], Zhao et al. showed Emi1 stabilizes SKP2 by inhibiting APC/C activity, accelerating p27 degradation and driving HCC progression [14], and Huang et al. reported hesperetin extracts inhibit proliferation via the SKP2-p27 axis [26]. In the present study, we found that curcumol reduced the mRNA and protein levels of SKP2, disrupted SKP2-p27 interactions, decreased p27 ubiquitination, and extended the half-life of p27, resulting in a marked increase in p27 abundance. Notably, SKP2 overexpression partially reversed these effects, mitigating curcumol-induced G1 arrest and growth suppression. Collectively, these data indicate that curcumol suppresses SKP2, leading to the accumulation and stabilization of p27, which ultimately triggers G1 phase arrest and inhibits the growth of SK-Hep-1 cells (Figure 6).

Recent evidence indicates that curcumol enhances APC/C-Cdh1 binding to SKP2 in colon cancer cells, activating its E3 ubiquitin ligase function and promoting SKP2 degradation [27]. E2F1 directly binds the SKP2 promoter, regulating SKP2 transcription and expression [28], suggesting multiple layers of SKP2 regulation. Studies have shown that PLK1 and Cdk1 phosphorylate Emi1, promoting its ubiquitination in vitro [29], and that PLK1 phosphorylates Emi1 during mitosis to trigger its degradation, activating APC/C [30]. Zhao et al. reported that Emi1 stabilizes SKP2 by inhibiting APC/C [14]. Combined with our previous finding [9], we propose that curcumol may modulate the Emi1-SKP2-p27 axis via PLK1 to induce G1 arrest. However, further study is needed to investigate the interactions of PLK1-SKP2 and their roles in HCC cell cycle progression. Additionally, as an F-box protein within the SCF complex, SKP2 relies on its F-box domain for E3 ligase activity [31], while its recognition of p27 depends on CKS1 [31,32]. Thus, the impact of curcumol on SKP2-p27 interactions also needs deeper exploration.

Finally, different tumour cells may exhibit varying sensitivities to curcumol [33]. This study used only SK-Hep-1 cells for in vitro experiments; further validation is needed across multiple HCC cell models to confirm curcumin’s ability to inhibit hepatocellular carcinoma cell cycle progression via the SKP2-p27 axis.

## 4. Materials and Methods

### 4.1. Materials

Curcumol (purity > 98%) was obtained from the National Institute for the Control of Pharmaceutical and Biological Products (Beijing, China, Cat No. C100409). The compound’s identity and purity were verified by the supplier using high-performance liquid chromatography (HPLC) analysis, according to their standard quality control protocol. Curcumol was dissolved in anhydrous ethanol (XiLONG SCIENTIFIC, Shantou, China) for experiments. An equivalent volume of ethanol was added to control cells as a vehicle control (Con). The final ethanol concentration was adjusted to match that of the highest curcumol-treated group. Dulbecco’s Modified Eagle’s Medium (DMEM) and fetal bovine serum (FBS) were obtained from Gibco Life Technologies (Grand Island, NE, USA). MTT and penicillin–streptomycin were purchased from Solarbio Biotechnology Company (Shanghai, China). The Reverse Transcription Kit (with dsDNase) was acquired from Biosharp Life Sciences (Hefei, China), while SYBR Green qPCR Master Mix was sourced from GLPBIO (Montclair, CA, USA). The BCA assay kit and RIPA lysis buffer were obtained from Beyotime Biotechnology (Shanghai, China). MG132 (Cat. No. HY-13259) and Cycloheximide (CHX, Cat. No. HY-12320) were purchased from MedChemExpress (Monmouth Junction, NJ, USA). Antibodies against CDK6 and Cyclin D1 were purchased from ZEN-BIOSCIENCE (Chengdu, Sichuan, China); SKP2 from Affinity Biosciences (Changzhou, China); CDK4 from ImmunoWay Biotechnology Company (Suzhou, China); P27kip 1 from ProMab Biotechnologies, Inc. (Richmond, CA, USA); and ubiquitin (Ub, WL01368) from WanLei-BIO (Shenyang, China). Anti-rabbit IgG and anti-mouse IgG secondary antibodies were obtained from ZSGB-BIO (Beijing, China).

### 4.2. Cell Culture and Treatment

SK-Hep-1 cells were purchased from were from the Cell Bank, Chinese Academy of Sciences (Shanghai, China). 293T cells were obtained from the institute of clinic pharmacology of Xiangya Hospital. (Changsha, China). SK-Hep-1 and 293T cells were cultured in DMEM supplemented with 10% FBS and 1% penicillin–streptomycin at 37 °C in a 5% CO_2_ incubator.

### 4.3. MTT Assay

Overall, 100 μL of SK-Hep-1 cells (4.0 × 10^4^ cells per mL) was seeded overnight in a 96-well plate. Next, the cells were exposed to different concentrations of curcumol (0 µM, 100 µM, 200 µM and 400 µM) for 24 and 48 h. Following treatment, 20 μL of MTT solution was added to each well and incubated for 4 h. The supernatant was then discarded, and 150 µL of DMSO was added to dissolve the formazan crystals. Absorbance at 490 nm was measured using a microplate reader (TECAN, Männedorf, Switzerland) to assess cell viability differences between curcumol-treated and control groups.

### 4.4. Colony Formation

After trypsin digestion, the cells were centrifuged and seeded at 800 cells per well in a 6-well plate. After 24 h, curcumol (0 µM, 100 µM, 200 µM and 400 µM) was added, with media changes every three days. After 12 days, cells were fixed with 4% paraformaldehyde, stained with 0.1% crystal violet, and air-dried. Finally, colonies were imaged using a camera and quantified with Image J 2.1.0.

### 4.5. Flow Cytometry for Cell Cycle Detection

After 48 h of curcumol treatment, SK-Hep-1 cells (5.0 × 10^5^ per 70-mm dish) were harvested by trypsinization and fixed in cold 70% ethanol dissolved in PBS. The suspension was incubated at −20 °C for at least 12 h, and then washed twice with cold PBS. Cells were treated with RNase A (250 µg/mL, 30 µL) and stained with propidium iodide (PI, 10 µL) in 300 µL PBS. Following a 30 min incubation at room temperature in the dark, cell cycle distribution was analyzed using a flow cytometer (Accuri C6 Plus, BD Biosciences, Franklin Lakes, NJ, USA). Data were processed with FlowJo v10.8.1 (BD Biosciences, USA) to determine the proportion of cells in each phase.

### 4.6. RNA Sequencing (RNA-Seq) and Proteomics

The RNA sequencing (RNA-seq) and proteomic data were generated in our previous study [9]. In the current study, integrated bioinformatic analyses were performed in R (version 4.3.0). Log2 fold change (log2FC) values from transcriptomic and proteomic datasets were integrated based on gene symbols. Genes were classified according to differential expression thresholds (|log2FC| ≥ 1), and statistical significance was defined as *p* < 0.05. Pearson correlation analysis was conducted to assess the concordance between transcriptomic and proteomic alterations. Gene Set Enrichment Analysis (GSEA) was performed based on the proteomic dataset using the clusterProfiler package in R 4.3.0. Gene symbols were converted to Entrez IDs using the org.Hs.eg.db annotation database. Proteins were ranked according to their log2FC values, and KEGG pathway enrichment analysis was conducted using the Homo sapiens database. The *p*-value cutoff was set at 0.05. Core enrichment genes from two biologically relevant KEGG pathways of interest were extracted from the GSEA results. Shared genes between these pathways were identified by intersecting their respective core enrichment gene sets. These common genes were highlighted in the cnetplot network visualization to illustrate potential key regulators involved in overlapping biological processes. The raw sequencing data are available from the corresponding author upon reasonable request.

### 4.7. Cell Transfection

SK-Hep-1 cells were trypsinized and seeded into 6-well plates in DMEM with 10% FBS (no antibiotics), reaching 65% confluence the next day. After confirming proper attachment, cells were washed with PBS and infected with lentivirus in the presence of polybrene for 8 h at 37 °C. The medium was then replaced with fresh DMEM containing 10% FBS. After 48 h, GFP fluorescence was examined to evaluate infection efficiency. Surviving cells were expanded, collected, and analyzed by quantitative reverse transcription polymerase chain reaction (qRT-PCR) and Western blot to confirm SKP2 overexpression (OE), generating a stable SKP2-overexpressing SK-Hep-1 cell line.

### 4.8. Co-Immunoprecipitation (Co-IP)

To investigate the interaction and ubiquitination of p27, Co-IP assays were performed in SK-Hep-1 cells. For the interaction study, SK-Hep-1 cells were transfected with Flag-SKP2 using Lipofectamine 3000. After 48 h, cells were lysed in IP lysis buffer and centrifuged at 12,000 *g* for 15 min at 4 °C. Protein A/G Magnetic Beads (MCE) were pre-incubated with an anti-Flag antibody at 4 °C for 4 h, and then incubated with the lysate supernatants overnight at 4 °C. After five washes, bound proteins were eluted in SDS loading buffer and analyzed by Western blot.

For the ubiquitination study, SK-Hep-1 cells were treated with MG-132 (10 μM) for 6 h to inhibit proteasomal degradation. Cells were lysed in IP lysis buffer and centrifuged at 12,000 *g* for 15 min at 4 °C. Protein A/G Magnetic Beads (MCE) were pre-incubated with p27 antibody at 4 °C for 4 h, then incubated with the lysate supernatants overnight at 4 °C. After five washes, bound proteins were eluted in SDS loading buffer and analyzed by Western blot.

### 4.9. Tumor Xenograft Experiment

The in vivo xenograft study was conducted between November 2024 and December 2024. According to our prior research [9], 8 × 10^6^ SK-Hep-1 cells were subcutaneously injected into the left flank of 4-5-week-old male BALB/c nude mice (purchased from Hunan SJA Laboratory Animal Co., Ltd., Changsha, China). The mice were randomized into four groups (*n* = 6): Vector-Con, Vector-Cur (80 mg/kg), SKP2 OE-Con, and SKP2 OE-Cur (80 mg/kg). Upon reaching a tumor volume of approximately 50 mm^3^, control groups received the vehicle (absolute ethanol: propylene glycol: PBS = 2:2:1), while treatment groups received curcumol (80 mg/kg) daily via oral gavage. Tumor volume was measured every two days using the formula V = (length × width^2^)/2. After 16 days of treatment, the tumor volume reached 1500 mm^3^, and the mice were euthanized by overdose of the anesthetic. Tumor tissue samples were harvested and fixed in 4% paraformaldehyde for histological analysis. All animal procedures were approved by the Institutional Animal Care and Use Committee (IACUC) of Guilin Medical University (approval No. GLMC-IACUC-20241101) and were conducted in accordance with the Guidelines for the Care and Use of Laboratory Animals set forth by the university.

### 4.10. qRT-PCR Assay

Total RNA was extracted from SK-Hep-1 cells with Trizol (TIANGEN, Beijing, China), and then reverse transcribed into cDNA using a cDNA synthesis kit (Biosharp, Beijing, China). The resulting cDNA was then subjected to real-time PCR analysis using an ABI 7500 Fast system (Thermo Fisher, Waltham, MA, USA) with SYBR Green fluorescent dye. β-actin was used as an internal reference, relative gene expression was calculated via the 2^−ΔΔCt^ method. Primer sequences are provided in Table 1.

### 4.11. Immunoblotting (IB)

SK-Hep-1 cells and tumor tissue samples were collected, lysed in RIPA lysis buffer (4 °C) for 30 min, then centrifuged at 12,000 rpm for 20 min at 4 °C. Protein concentrations of both the cell and tumor tissue lysates were determined using a BCA protein assay kit (Beyotime Biotechnology, Shanghai, China). For Western blot analysis, equal amounts of protein were separated by SDS-PAGE and transferred onto membranes. The membranes were blocked and incubated with the appropriate primary and secondary antibodies. Protein bands were visualized using ECL reagents on a chemiluminescence imaging system (BIO-RAD, Hercules, CA, USA), and band grayscale values were quantified and statistically analyzed using Image J 2.1.0.

### 4.12. Immunohistochemistry (IHC) Staining

Tumor tissue was fixed in formalin and embedded in paraffin. Tissue sections were dewaxed and rehydrated following baking at 60 °C for 30 min, then deparaffinized and rehydrated through xylene and a graded ethanol series to distilled water. For antigen retrieval, sections were immersed in citrate buffer (pH 6.0) and heated in a water bath at 92 °C for 40 min, with the temperature maintained and the liquid level monitored to ensure submersion, before cooling naturally to room temperature. To block endogenous peroxidase activity, sections were incubated in freshly prepared 3% hydrogen peroxide (H_2_O_2_) for 10 min at room temperature, followed by three 5-min washes with deionized water. Nonspecific binding was minimized by blocking sections in 5% BSA (diluted in PBS) at 37 °C for 30 min. Without washing, primary antibodies against SKP2 and p27, diluted to working concentrations in 5% BSA, were applied to the sections and incubated overnight at 4 °C in a humidified chamber to prevent evaporation. The next day, sections were washed three times with PBS (5 min each), ensuring complete submersion in fresh PBS each time. HRP-conjugated secondary antibody was then added, and sections were incubated for 60 min at room temperature in the dark. After three additional PBS washes, DAB substrate was applied for color development, followed by rinsing with deionized water to stop the reaction and counterstaining with hematoxylin. Sections were dehydrated through a graded ethanol series to xylene, mounted with neutral resin, and observed under a microscope once the resin had fully dried.

### 4.13. Statistical Analysis

Statistical analyses were performed using SPSS version 20.0. Data were expressed as mean ± standard deviation (SD). Two-sample comparisons employed independent *t*-tests, while multi-group variances were assessed via one-way ANOVA. Kruskal–Wallis one-way analysis of variance was employed for non-normally distributed data or those not satisfying variance chisquare. Significance was denoted by * *p* < 0.05, ** *p* < 0.01, ^#^ *p* < 0.05 and ^##^ *p* < 0.01.

## 5. Conclusions

In the study, the natural compound curcumol modulated the SKP2-p27 axis by reducing the interaction between p27 and SKP2. This leads to stabilization and increased levels of p27 protein, resulting in G1 phase arrest. These findings revealed a novel antitumor mechanism of curcumol, suggesting it could be a promising agent for HCC therapy via the SKP2-p27 axis.

## Figures and Tables

**Table 1:** Sequences of forward and reverse primers used in qRT-PCR.

RNA Species	Primer Pairs
*Skp2*	Forward: 5′-CCCAATCTTGTCCATCTAGACTT-3′
	Reverse: 5′-CATAGCACCGACTGAGTGATAG-3′
*β-actin*	Forward: 5′-AAAGACCTGTACGCCAACAC-3′
	Reverse: 5′-GTCATACTCCTGCTTGCTGAT-3′

## Data Availability

Data will be made available upon request from the corresponding authors.
